# Detecting PM2.5’s Correlations between Neighboring Cities Using a Time-Lagged Cross-Correlation Coefficient

**DOI:** 10.1038/s41598-017-10419-6

**Published:** 2017-08-31

**Authors:** Fang Wang, Lin Wang, Yuming Chen

**Affiliations:** 1 College of Science/Agricultural Mathematical Modeling and Data Processing Center, Hunan Agricultural University, Changsha, P. R. China; 2 Department of Mathematics and Statistics, University of New Brunswick, Fredericton, NB E3B 5A3 Canada; 3 Department of Mathematics, Wilfrid Laurier University, Waterloo, ON N2L 3C5 Canada

## Abstract

In order to investigate the time-dependent cross-correlations of fine particulate (PM2.5) series among neighboring cities in Northern China, in this paper, we propose a new cross-correlation coefficient, the time-lagged *q-L* dependent height crosscorrelation coefficient (denoted by *p*_*q*_(*τ*, *L*)), which incorporates the time-lag factor and the fluctuation amplitude information into the analogous height cross-correlation analysis coefficient. Numerical tests are performed to illustrate that the newly proposed coefficient *ρ*_*q*_(*τ*, *L*) can be used to detect cross-correlations between two series with time lags and to identify different range of fluctuations at which two series possess cross-correlations. Applying the new coefficient to analyze the time-dependent cross-correlations of PM2.5 series between Beijing and the three neighboring cities of Tianjin, Zhangjiakou, and Baoding, we find that time lags between the PM2.5 series with larger fluctuations are longer than those between PM2.5 series withsmaller fluctuations. Our analysis also shows that cross-correlations between the PM2.5 series of two neighboring cities are significant and the time lags between two PM2.5 series of neighboring cities are significantly non-zero. These findings providenew scientific support on the view that air pollution in neighboring cities can affect one another not simultaneously but with a time lag.

## Introduction

As a cost to rapid development of economics and progress of technology after World War II, the environmental pollution, particularly air pollution produced by industry exhaust, smoke dust, and coal combustion, has become a serious world-wide problem^1^. For instance, the Great Smog of 1952 took away 4,000 lives and led to more than 100,000 people affected by respiratory diseases, which has been regarded as the most serious environmental disaster in British history^2^. Urban air pollution directly and indirectly caused by rapid urbanization and industrialization in China in the last three decades has become more and more severe. Despite the decrease in “traditional pollutants” (e.g. NO2, SO2), fine particulate matter with a diameter ≤2.5 *μm* (PM2.5) has become a major air pollutant that threatens human’s health, including morbidity and mortality, and decreases meteorological visibility^3–13^. Naturally, as a hot societal issue, study on air pollution has attracted enormous attention from researchers in economical modeling^3–8^, as well as statistical modeling^9–13^. With the advancement of modern statistic methods, it is of great importance to assess smog’s trend and propagation characteristics from statistical point of view. In this context, much of existing work in the literature has been focused on studying the correlations among various air pollution indicators such as, air pollution index (API), air quality index (AQI), PM2.5 concentrations, PM10 (particulate with a diameter ≤10 *μm*) concentrations, temperature, air pressure, rainfall, relative humidity, and wind speed^9–13^. It is a common sense that smog produced at one source place can spread to its surrounding areas^6, 7^. Hence, it is more practical to explore cross-correlations of the above air pollution indicators among neighbouring cities as this helps the authority further assess the causes of local smog.

To study cross-correlations between two series, several statistical methods have been proposed in the literature. The methods include the widely used detrended cross-correlation analysis (DCCA)^14, 15^, and its multifractal version (MF-DXA)^16^, detrended moving average cross-correlation analysis (DMXA)^17^ and MF-DMXA^18^, multifractal cross-correlation analysis (MFCCA)^19^, multifractal height cross-correlation analysis (MF-HXA)^20^ and its extension the analogous MF-HXA (AMF-HXA)^21^. Later, Podobnik *et al*.^22, 23^ developed some statistical tests to test the presence of power-law cross-correlation between series. Moreover, DCCA with partial-correlation technique has been shown to be a powerful tool to reveal the intrinsic correlations between the two studied series interfered by another identical series^24–26^. Associated with the above mentioned methods, several cross-correlation coefficients were proposed naturally^21, 25–33^. However, the majority of the existing work focused mainly on the synchronous time series while only limited work considered the cross-correlations between two series with time lags^34–37^. In addition, the limited existing work in^34–36^, was established on the basis of the detrended fluctuation analysis (DFA)^38, 39^, and DCCA^14, 15^. The so-called time-lagged DCCA cross-correlation coefficient was proposed by Shen *et al*.^36^, which can be used to detect time-dependent cross-correlations between the API and wind speed but fails to recognize the range of fluctuation amplitudes that contributed to those cross-correlations. The *q*-dependent detrended cross-correlation coefficient proposed by Kwapień *et al*.^33^ has the flexibility to successfully detect range of cross-correlated fluctuations, but it is not applicable to series that are correlated with time lags. In this work, inspired by the paper^33^, we propose a time-lagged cross-correlation coefficient that can be used to identify the range of detrended fluctuation amplitudes for signals correlated with time lags.

**Figure 1:**
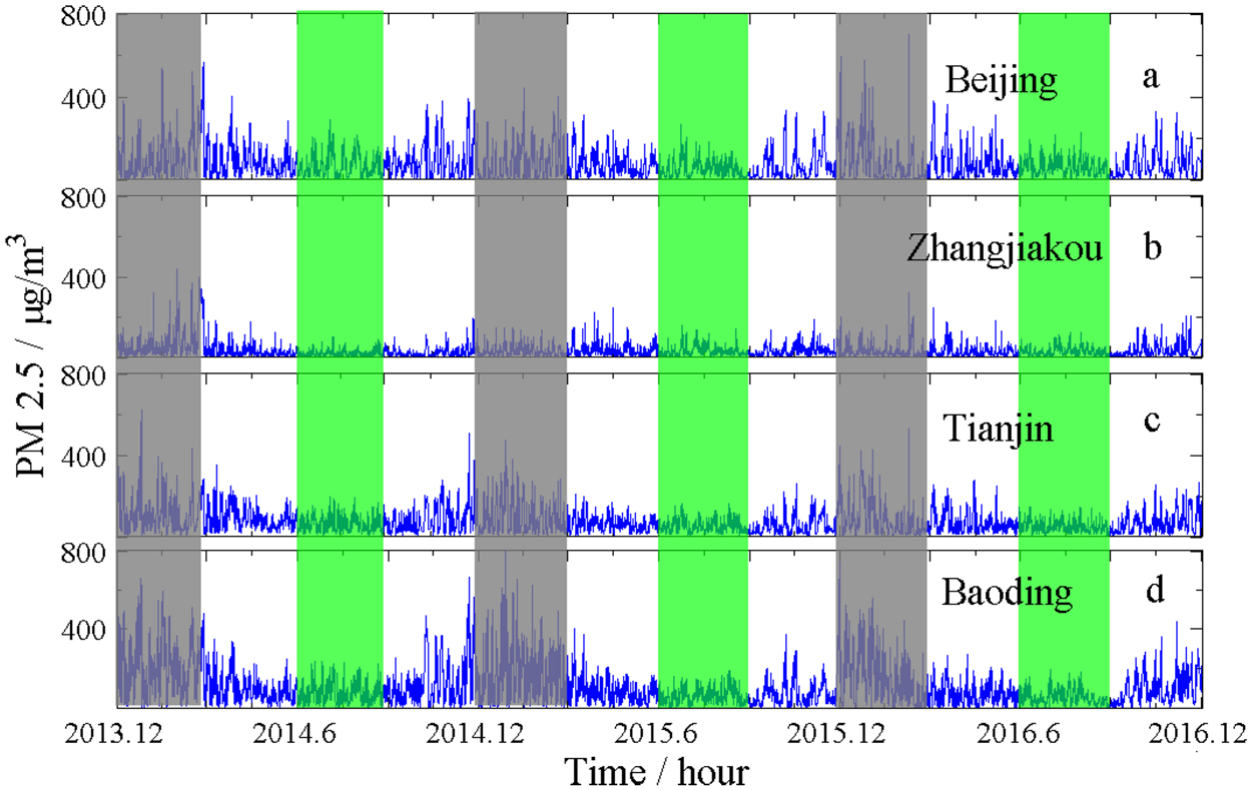
PM2.5 series of the four chosen cities in Northern China during the period of Dec. 1, 2013 and Nov. 30, 2016. The gray area refers to the winter season and the green area refers to the summer season of every year.

Another motivation of this work is to explore if there are cross-correlations for the PM2.5 series between neighbouring cities. In this study, we focus our interest on the interaction of PM2.5 series of four cities in Beijing-Tianjin-Hebei (known as the Jing-Jin-Ji area) of China, namely, Beijing (39.93°*N*,116.39°*E*), Zhangjiakou(40.81°*N*,114.89°*E*), Tianjin (39.14°*N*,117.21°*E*), and Baoding (38.89°*N*,115.49°*E*). Zhangjiakou, Tianjin, and Baoding are surrounding Beijing and are about 160 km, 115 km, and 140 km away from Beijing, respectively. All four cities have been greatly affected by heavy smog in recent years. Real-time data of PM2.5 series of these four cities from December 1, 2013 to November 30, 2016 are chosen for our consideration. The data are taken from the Ministry of Environmental Protection of the People’s Republic of China (http://datacenter.mep.gov.cn) and are shown in Fig. 1.

Figure 1 clearly demonstrates that there is a huge difference in the PM2.5 concentrations between the summer and the winter. We recorded hourly data of severe pollution (PM2.5 concentration > 150 *μg*/*m* ^3^) of the four cities in the whole studied time period. There are 1427, 272, 1439, and 3338 hours in the three winters, respectively, while there are only 303, 2, 58, and 279 hours in the three summers, respectively. We also note in Fig. 1 that PM2.5 concentrations in the four cities follow similar trend. This indicates that cross-correlations among these series may exist.

To fully detect and quantify possible cross-correlations among the PM2.5 series mentioned above and uncover the potential time lags embedded in those cross-correlations, in this work, we propose a new cross-correlation coefficient by incorporating the time-lag factor and fluctuation information into the latest analogous height cross-correlation analysis (AHXA) coefficient *ρ*(*L*) introduced by Wang *et al*.^21^. The new coefficient, denoted by *ρ*_*q*_(*τ*, *L*), will be referred to as the time-lagged *q*-*L* dependent height cross-correlation coefficient. Two numerical tests will be performed to assess the performance of the proposed *ρ*_*q*_(*τ*, *L*). The first test is used to illustrate that the new coefficient *ρ*_*q*_(*τ*, *L*) can accurately detect the cross-correlation between two series with certain time lags, while the second test is to show that the new coefficient is capable of distinguishing correlations between small and large fluctuation amplitudes. Applying the new coefficient to analyze the PM2.5 series recorded in Fig. 1 helps us understand the spreading characteristics of PM2.5 among neighboring cities in Northern China.

## Results and Discussions

### Performance of *ρ*_*q*_(*τ,L*)

The time-lagged *q*-*L* dependent AHXA coefficient *ρ*_*q*_(*τ*,*L*) incorporates both time-lagged covariance function and the *q*-th order (co-)variance function. The former allows us to detect correlations between two series asynchronously while the latter makes the coefficient flexible to detect the range of cross-correlated fluctuations. In this section, we perform two numerical tests to illustrate the advantages and applicability of the proposed coefficient *ρ*_*q*_(*τ*,*L*).

**Figure 2:**
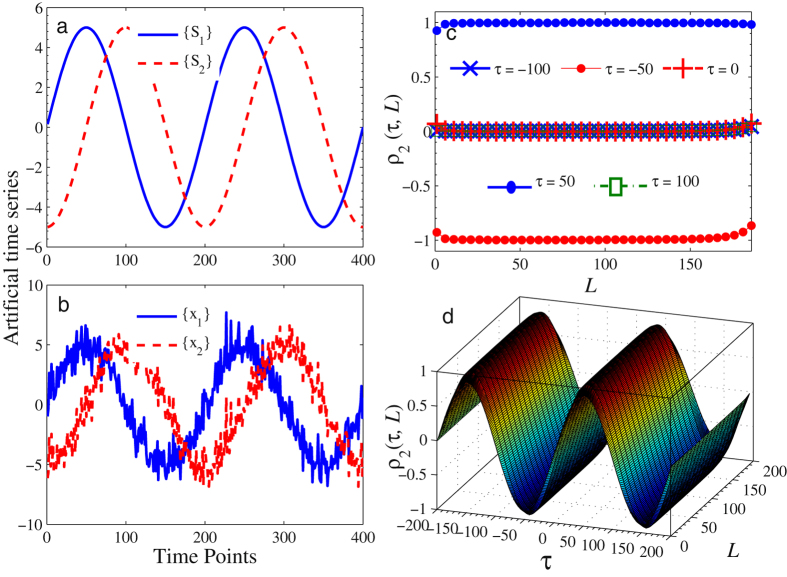
Numerical results of Test 1. (**a**) Sinusoidal signals *S*_1_ and *S*_2_; (**b**) Time series {*x*_1_} and {*x*_2_} generated by two sinusoidal signals with Gaussian noises; (**c**) Calculated time-lagged cross-correlation coefficients *ρ*_2_(*τ*,*L*); (**d**) Calculated *ρ*_2_(*τ*,*L*) surface.

In our first numerical test, we generate a pair of artificial time series *x*_1_(*t*) = *S*_1_(*t*) + *ε*(*t*) and *x*_2_(*t*) = *S*_2_(*t*) + *ε*(*t*) for *t* = 1, 2, …, 10,000, where {*ε*(*t*)} is a white noise; {*S*_1_} and {*S*_2_} are two sinusoidal signals with the same period *p* = 200 and the same amplitude but different phases (they differ by one quarter of the period, as shown in Fig. 2a). Thus, {*x*_1_(*t*)} and {*x*_2_(*t*)} are two periodic sequences having the same noises (see Fig. 2b). Theoretically, the two series {*x*_1_(*t*)} and {*x*_2_(*t*)} should be perfectly correlated. But because of the difference in phases, the corresponding Pearson correlation coefficient is calculated to be 0.075 (very close to 0), which fails to detect any correlation between the two series {*x*_1_(*t*)} and {*x*_2_(*t*)}. However, we can calculate the time-lagged cross-correlation coefficients by setting *τ* as −100, −50, 0, 50 and 100, respectively. The obtained results with *q* = 2 are shown in Fig. 2c. As can be seen in Fig. 2c, when *τ* takes values −100, 0 and 100, the resulting coefficients are almost 0 and when $\tau =\frac{p}{4}=50$, the two series exhibit perfect positive correlations. Perfect negative correlations are observed when $\tau =-\frac{p}{4}=-50$. Figure 2d depicts the proposed time-dependent correlation coefficients (when *q* = 2) as the time-delay *τ* varies from −200 to 200.

Our second numerical test aims to show that the new coefficient *ρ*_*q*_(*τ*,*L*) is capable of identifying different correlations at different fluctuation amplitudes. To this end, binomial multifractal series (BMFs), which can produce more different degrees of power-law for different fluctuation amplitudes, is employed in our test. Two series of {*y*_1_} and {*y*_2_} are constructed as

1 $${y}_{i}={p}_{i}^{n-n[k-1]}{(1-{p}_{i})}^{n[k-1]},\quad k=1,\,2,\ldots ,\,{2}^{n},\quad i=1,\,2.$$

Here parameters *p*_*i*_ ∈ (0, 0.5) (*i* = 1, 2), *n*[*k*] denotes the number of digit 1 in the binary representation of the index *k*. In this test, we set *p*_1_ = 0.3 and *p*_2_ = 0.4. Each series is of length 2^15^. To illustrate that the proposed *ρ*_*q*_(*τ*,*L*) can provide more information about cross-correlations at different fluctuation amplitudes, the following two experiments are carried out.

**Figure 3:**
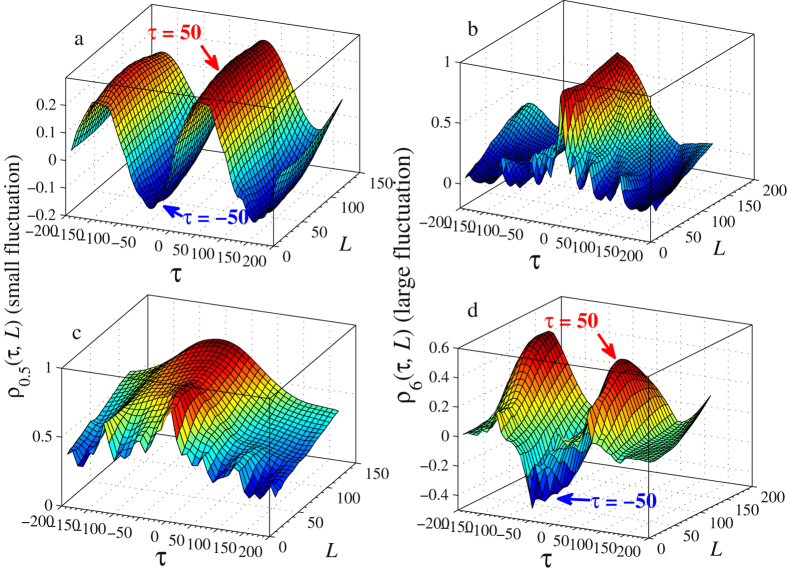
Calculated coefficients *ρ*_*q*_(*τ*,*L*) for two BMFs with *p*_1_ = 0.3 and *p*_2_ = 0.4. (**a** and **b**) Show the *ρ*_*q*_(*τ*,*L*) surface when *q* = 0.5 and 6, which describe the cross-correlation between the compound series with dominated small fluctuations and large fluctuations, respectively. (**c** and **d**) Are the opposite of (**a** and **b**), respectively.

In Experiment I, we first locate the data points with small amplitudes satisfying *y*_1_ < 9 × 10^−7^ and *y*_2_ < 10^−4^, we then replace those data points by two sinusoidal signals *G*_1_ = 0.0004 sin $(\frac{2\pi x}{200})$ and *G*_2_ = 0.00004 sin $(\frac{2\pi x}{200}-\frac{\pi }{2})$ accordingly. In this way, two new series {*y*′_1_} and {*y*′_2_} are generated. Since the data in the original series with small fluctuations are replaced by the two sinusoidal signals, the correlation between the new series {*y*′_1_} and {*y*′_2_} at small fluctuations is dependent on {*G*_1_} and {*G*_2_} while the correlation preserves among large fluctuations. Numerical results are presented in Fig. 3a and b. In Fig. 3a, *q* = 0.5 refers to small fluctuation amplitude. The calculated *ρ*_0.5_(*τ*,*L*) surface exhibits periodicity with period of 200, the same as the two sinusoidal signals. The maximum of positive correlation coefficient reaches at *τ* = 50, which is exactly the phase difference between {*G*_1_} and {*G*_2_}. On the contrary, the maximum negative correlation coefficient appears at *τ* =  −50, which means the two series are exactly half a period apart. Figure 3b illustrates the results for large fluctuation amplitudes with *q* = 6. As expected, the shape of the *ρ*_6_(*τ*,*L*) surface is not affected by the periodic signals, thus, the maximum correlation coefficient is obtained when there is no time lag between the {*y*′_1_} and {*y*′_2_}, i.e., *τ* = 0. In Experiment II, We remove from the series {*y*_1_} all values which are larger than 5 × 10^−4^ and from the series {*y*_2_} those values larger than 10^−4^, and replace corresponding values by *G*_3_ = 0.004 sin $(\frac{2\pi x}{200})$ and *G*_4_ = 0.0004 sin $(\frac{2\pi x}{200}-\frac{\pi }{2})$, respectively. Consequently, for the obtained new series, the cross-correlation at relative large fluctuations depends on the series {*G*_3_} and {*G*_4_} but the correlation preserves among the small fluctuations. Associated numerical results are presented in Fig. 3c and d. As can be seen from Fig. 3d, the maximum positive correlation coefficient appears at *τ* = 50 and *τ* =  −150 and the maximum negative correlation coefficient occurs when *τ* =  −50. These two experiments clearly illustrate that the proposed time-lagged *q*-*L* dependent AHXA coefficient *ρ*_*q*_(*τ*,*L*) can capture not only fluctuation information but also potential time-delay in cross-correlations.

### Application of *ρ*_*q*_(*τ*,*L*) to PM 2.5 series

In this section, we apply our time-lagged cross-correlation coefficient *ρ*_*q*_(*τ*,*L*) to the PM2.5 series mentioned in the introduction to illustrate the applicability of *ρ*_*q*_(*τ*,*L*). Three factors, namely, the season factor in Northern China, the fluctuation amplitudes of PM2.5, and the time interval used to calculate *ρ*_*q*_(*τ*,*L*), are considered to explore the time-lagged correlations. For the data reported in Fig. 1, four seasons are classified as winter (December, January, and February), spring (March, April, and May), summer (June, July, and August), and fall (September, October, and November). Two representative fluctuation amplitudes, i.e., small and large fluctuations, characterized by *q* = 0.5 and *q* = 6, respectively, are set for our consideration. The time interval *L* is set to range from 24 hours to 720 hours with the step size 24. This means we only focus on the correlation of each series at day 1, day 2, up to day 30.

**Figure 4:**
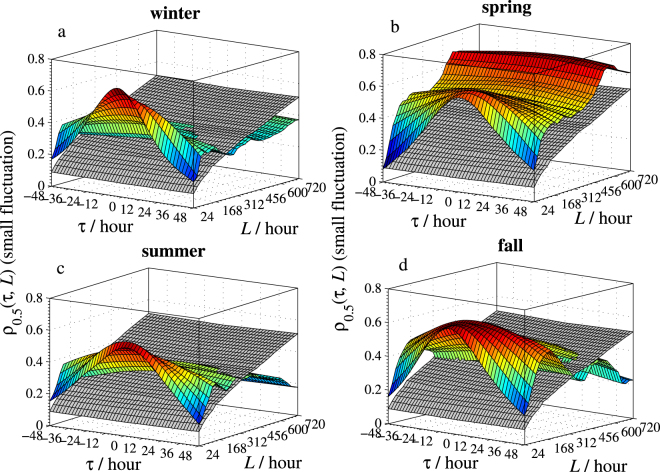
The time-lagged cross-correlation coefficient *ρ*_*q*_(*τ*,*L*) with *q* = 0.5 of PM2.5 series between Beijing and Zhangjiakou. The threshold surfaces are colored in gray.

**Figure 5:**
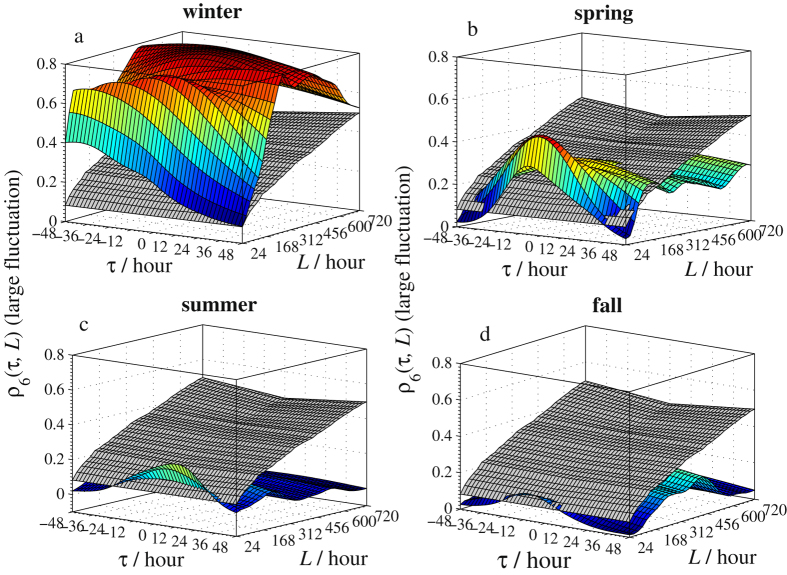
The time-lagged cross-correlation coefficient *ρ*_*q*_(*τ*,*L*) with *q* = 6 of PM2.5 series between Beijing and Zhangjiakou. The threshold surfaces are colored in gray.

The calculated *ρ*_*q*_(*τ*,*L*) surfaces of Beijing vs. Zhangjiakou for the four seasons with the two selected *q*’s are sketched in Figs 4 and 5. To help determine if the cross-correlation between PM2.5 series in the two cities is significant, threshold surfaces ${\rho }_{q}^{c}(\tau ,L)$ (colored in gray in those sub-figures) are also constructed in Figs 4 and 5 for each season and both *q*’s. These threshold surfaces are numerically constructed via the Monte-Carlo simulations proposed by Podobnik *et al*.^23^, where we used Gaussian series and repeated the calculations for 10,000 times at the 95% significance level. Note that ${\rho }_{q}(\tau ,L) > {\rho }_{q}^{c}(\tau ,L)$ means that the cross-correlation is significant at the point (*τ*,*L*) and vice versa. For the small fluctuation amplitudes of original series (refers to *q* = 0.5, Fig. 4), except for the spring, the cross-correlations vary from small time intervals to large time intervals in all other three seasons. More specifically, the *ρ*_0.5_(*τ*,*L*) surfaces are above the threshold surfaces ${\rho }_{q}^{c}(\tau ,L)$ for *L* < 288*h* (12d) ∼408 *h* (17d) approximately. This implies that the long-range correlation at small fluctuation amplitudes lasts up to 12∼17 days. This interesting finding explains that the PM2.5 of a relatively stable range in Beijing and Zhangjiakou can affect each other and the interaction will continue for almost 17 days in winter, summer, and fall. Meanwhile, in spring, this interaction lasts longer. However, as seen from Fig. 5, for the cross-correlation of PM2.5 with larger fluctuations (refers to *q* = 6), the spreading is most significant in winter but is almost absent in summer and fall. This indicates that the smog spread between Beijing and Zhangjiakou is most serious in winter and is least serious in summer and fall. In addition, the interaction of heavy smog between Beijing and Zhangjiakou lasts about 312 *h* (13d) in spring, which is much less than the interaction time in winter. Similar patterns are also observed for the PM2.5 series of Beijing vs. Tianjin and Beijing vs. Baoding: the long-range correlation at small fluctuation amplitudes lasts up to 15∼19 days in the winter and summer between Beijing and Tianjin while that lasts more days in spring and fall. Further, the long-range correlation at large fluctuation amplitudes can be found in all studied days in all seasons except for fall, while the correlation lasts for only 19 days in fall. For Beijing vs. Baoding: the long-range correlation at small fluctuation amplitudes lasts up to 22∼29 days in the winter and fall, and all observed days in spring and summer. Meanwhile, the long-range correlation at large fluctuation amplitudes lasts longer in spring and summer than in winter and fall.

**Figure 6:**
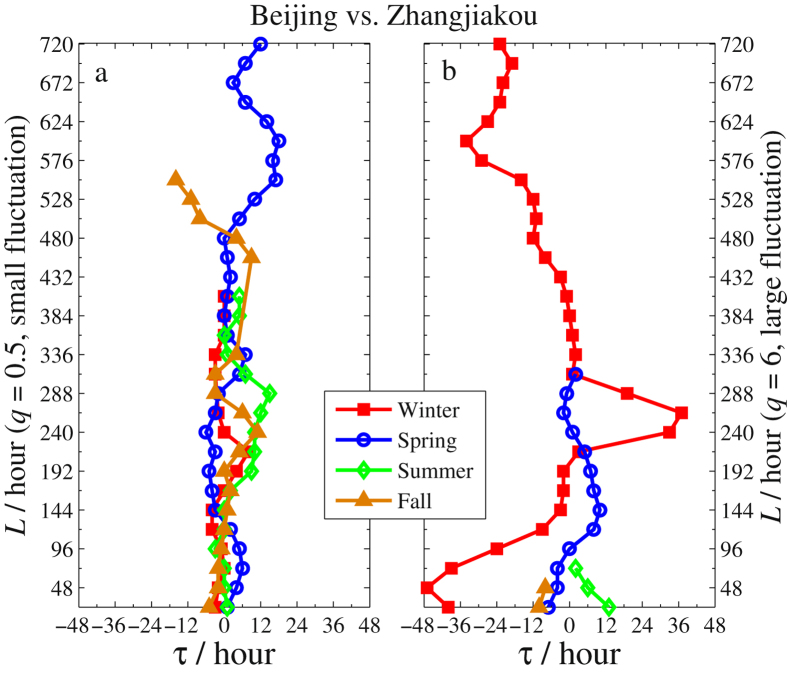
The *τ*-*L* curves of Beijing vs. Zhangjiakou in four seasons. (**a**) Is for the PM2.5 with small fluctuations (*q* = 0.5) and (**b**) is for the PM2.5 with large fluctuations (*q* = 6).

**Figure 7:**
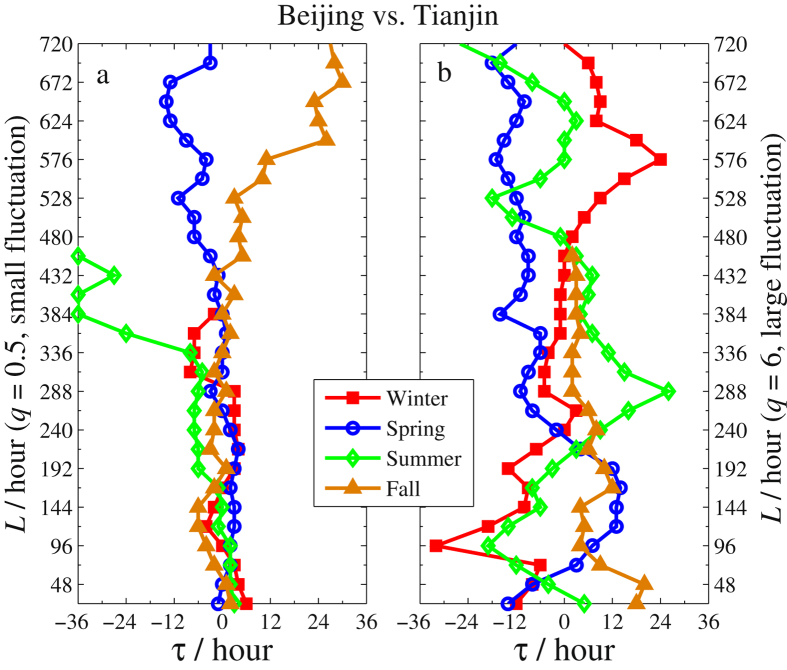
The *τ*-*L* curves of Beijing vs. Tianjin in four seasons (The legends are the same as in Fig. 6).

**Figure 8:**
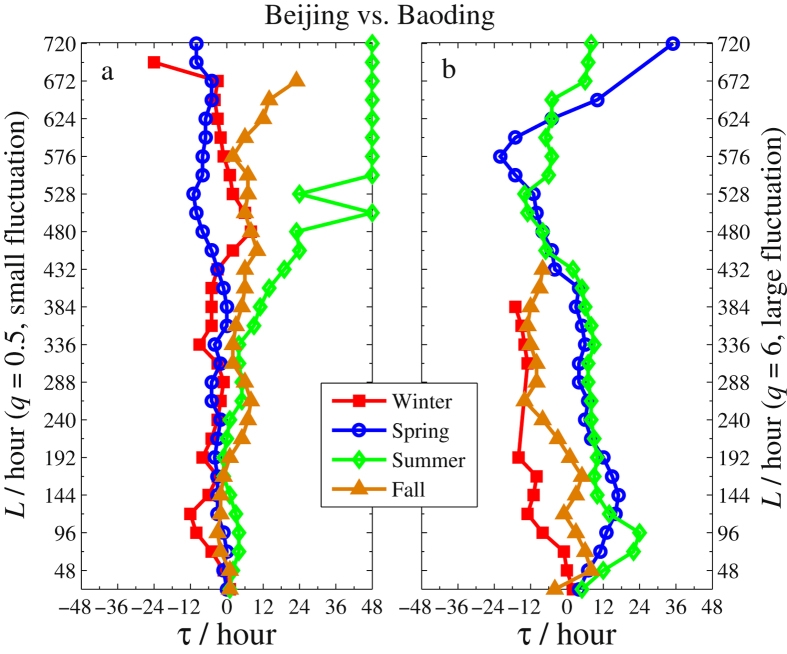
The *τ*-*L* curves of Beijing vs. Baoding in four seasons (The legends are the same as in Fig. 6).

Besides the difference due to the season factor and the fluctuation amplitudes acquired from the eight sub-figures, one can also obtain information about the time lags of PM2.5 series between two neighbouring cities. For illustration, we sketch the *τ*-*L* curves in Figs 6–8 for Beijing vs. Zhangjiakou, Beijing vs. Tianjin, and Beijing vs. Baoding, respectively. In each figure, *τ* is the time lag corresponding to the maximum of *ρ*_*q*_(*τ*,*L*) for each *L*. When we record *τ* in calculating *ρ*_*q*_(*τ*,*L*), we use *x* to stand for PM2.5 series of Beijing and *y* for that of the other three cities. In this setting, *τ* > 0 indicates that the largest *ρ*_*q*_(*τ*,*L*) is obtained at the time point where Zhangjiakou’s (or Tianjin’s, or Baoding’s) PM2.5 lags are behind Beijing’s by *τ* hours. This implies that Beijing’s air pollution will affect the other three cities. Conversely, *τ* < 0 means that Beijing’s air pollution will be affected by the other three cities. Here we only report these *τ*’s obtained by the largest cross-correlation that is significant (${\rho }_{q}(\tau ,L)\ge {\rho }_{q}^{c}(\tau ,L)$) in each figure. In addition, to eliminate the influence of the time intervals *L*, we average the *ρ*_*q*_(*τ*,*L*) over every *L* and record the maximum together with its corresponding time lag *τ*. The results are listed in Table 1.

**Table 1:** The maximum of *ρ*_*q*_(*τ*,*L*) averaged over the intervals *L* and the corresponding time lag *τ* of Beijing vs. the three neighboring cities in four seasons.

Season	*q*	Beijing vs. Zhangjiakou		Beijing vs. Tianjin		Beijing vs. Baoding	
		*ρ*	*τ*	*ρ*	*τ*	*ρ*	*τ*
winter	0.5	0.3898*	−2	0.4533*	2	0.5373*	−3
	6	0.7181*	−10	0.4822*	−7	0.3184*	−6
spring	0.5	0.6085*	2	0.7211*	0	0.7586*	−2
	6	0.2925	−1	0.7456*	−4	0.5915*	7
summer	0.5	0.3576*	2	0.4725*	−1	0.6365*	5
	6	0.0795	8	0.7511*	−1	0.6590*	8
fall	0.5	0.4533*	−3	0.5913*	1	0.6216*	2
	6	0.1583	−3	0.4353*	9	0.3676*	−1

*Denotes 95% confidence level.

Based on Figs 6–8 and Table 1, we arrive at the following conclusions.

1. The time lags obtained by *ρ*_*q*_(*τ*,*L*) with larger fluctuation amplitudes (Figs 6b–8b) are longer than those with smaller fluctuation amplitudes (Figs 6 a–8a) and the range of the former is also much wider. This indicates that severe smog produced by PM2.5 in one city would slowly affect the air quality in its neighboring cities and the influence would last longer. For the PM2.5 of small fluctuation amplitudes, the cross-correlations between Beijing and the other three neighboring cities are significant for all four seasons and smog in those cities influences one another since *τ* fluctuates around 0. This explains that the air quality in one city of Northern China cannot be irrelevant to that of its neighbouring cities.
2. As shown in Fig. 6b and Table 1, most time lags are less than 0 in Beijing’s winters. This indicates that the air in Beijing was greatly influenced by the pollution from Zhangjiakou. In fact, due to the domination of the northwest wind from Siberia in Zhangjiakou’s winter, the pollution of Zhangjiakou (located in the northwest of Beijing) can easily spread to Beijing. Furthermore, there are about 50% of time during which the wind force is above level IV (the average wind speed is greater than 30 *km*/*h*) in Zhangjiakou’s winter, which drifts the polluted air to Beijing in less than 6 hours. This is consistent with the obtained lags for the significant cross-correlations between Zhangjiakou and Beijing. In addition, the cross-correlations of PM2.5 between Beijing and Zhangjiakou are insignificant in the other three seasons.
3. Another source of heavy air pollution in Beijing’s winter is Baoding since the associated time lag *τ* < 0 (see Fig. 8b). Nevertheless, compared to winter and fall, the smog spreads in spring and summer mainly from Beijing to Baoding since *τ* > 0 during most time intervals and the spread is quite slow. Indeed, as seen from Table 1, the time lag corresponding to the maximum of mean *ρ*_*q*_(*τ*,*L*) is 7–8 hours in spring and summer, and this number is 6 and 1 in winter and fall, respectively.
4. Compared to Zhangjiakou and Baoding, heavy air pollution in Beijing and Tianjin has a greater impact on each other. This is due to the closer distance between the two cities. In the most severe smog of winter, it is clearly seen from Fig. 7b that the time lag *τ* < 0 when the interval *L* starts at 24 h (1d) and lasts up to 360 h (15d) while *τ* > 0 when *L* is beyond 15 days. This shows that the heavy smog in a winter of Tianjin would invade Beijing at a short time scale but Tianjin would be invaded by Beijing’s heavy smog in a longer time scale. Overall, as shown in Table 1, except for the fall, the severe smog in the other three seasons spreads mainly from Tianjin to Beijing.

## Conclusions

In this work, by incorporating the time-lagged factor and the fluctuation amplitude information into the cross-correlation coefficient *ρ*(*L*) based on analogous height cross-correlation analysis, we have proposed the time-lagged *q*-*L* dependent AHXA coefficient *ρ*_*q*_(*τ*,*L*). This newly proposed cross-correlation coefficient can be used to quantify the range of cross-correlated fluctuations with more flexibility for two time series with potential time lags. It has been shown via two tests that this new coefficient (i) can accurately capture time-lagged cross-correlations hidden between two time series; (ii) can precisely locate the time points at which the studied two series are perfectly correlated; (iii) can successfully quantify different ranges of cross-correlations between two series with different fluctuation amplitudes.

We applied this new cross-correlation coefficient to study the PM2.5 series of four neighboring cities in Northern China and found that different time lags exist in the PM2.5 series of Beijing and the other three neighboring cities at different seasons and different fluctuation amplitudes. These findings provide very useful insights on understanding the pattern of smog’s propagation in Northern China and on accurately predicting future air pollution conditions. The idea can also be applied to many other fields for investigating time-dependent correlations. One particular field might be the new field of network physiology^40^. Recently, the concept of time delay stability (TDS) among key organ systems has been used to quantify physiologic interaction and transitions across distinct functional states^41, 42^. It has been found that each physiologic state is characterized by a distinct network structure with different relative contribution from individual organ systems to the global network dynamics^42^. This shows the cross-correlation is sensitive to fluctuation amplitudes and time scale. Thus the proposed method might be applied to probe complex coupling among networks of dynamical systems and to characterize the emergent functions.

## Methods

Incorporating the time lag operator into the method of analogous multifractal height cross-correlation analysis (AMF-HXA) developed by Wang *et al*.^21^, we derive our time-lagged *q*-*L* dependent AHXA coefficient in this section. For two given time series {*x*_*t*_} and {*y*_*t*_}, *t* = 1, 2, …, *N*, the associated accumulated deviation series are computed by $X(t)={\sum }_{i=1}^{t}[{x}_{i}-\langle x\rangle ]$, $Y(t)={\sum }_{i=1}^{t}[{y}_{i}-\langle y\rangle ]$, where 〈⋅〉 denotes the mean value of the whole time signal. The cross-increment of the two accumulated series with time interval *L* is defined by

2 $${{\rm{\Delta }}}_{L}X(t)Y(t+\tau )=[X(t)-X(t+L)]\cdot [Y(t+\tau )-Y(t+\tau +L)],$$

where *τ* takes any integer within the range of the series.

In order to record the average fluctuation of cross-increment between {*X*(*t*)} and {*Y*(*t* + *τ*)}, inspired by ref. 19 and ref. 21, we employ a symbolic operator to describe the real information of Δ_*L*_ *X*(*t*)*Y*(*t* + *τ*) for every *τ* and *L*. Hence, the *q*-th order covariance between the {*X*(*t*)} and {*Y*(*t* + *τ*)} can be determined by

3 $${F}_{xy}^{q}(L)=\langle {\rm{sign}}({{\rm{\Delta }}}_{L}X(t)Y(t+\tau ))\cdot |{{\rm{\Delta }}}_{L}X(t)Y(t+\tau {)|}^{q\mathrm{/2}}\rangle ,$$

where *t* varies from 1 − *τ* to *N* − *L* when *τ *< 0 and from 1 to *N* − *L* − *τ* when *τ* ≥ 0. The sign(Δ_*L*_ *X*(*t*)*Y*(*t* + *τ*)) denotes the sign of Δ_*L*_ *X*(*t*)*Y*(*t* + *τ*). The preserved sign information of Δ_*L*_ *X*(*t*)*Y*(*t* + *τ*) in ${F}_{xy}^{q}(L)$ provides *i*) the fluctuation information as well as the sign information of the fluctuation function; *ii*) correct cross-correlated exponent (if correlated), and *iii*) true correlation of {*x*(*t*)} and {*y*(*t*)} with time-delay *τ* avoiding spurious correlations. The ${F}_{xy}^{q}(L)$ expresses different fluctuations through the different order *q*, which describes smaller fluctuations with smaller *q*’s. In general, exponent *q* < 1 magnifies the contribution to ${F}_{xy}^{q}(L)$ from small amplitude fluctuations and *q* > 2 magnifies the contribution from large amplitude fluctuations. Besides the *q*-th order covariance, one can also obtain the *q*-th order variance for a single signal by letting *x*_*t*_ = *y*_*t*_ to arrive

4 $${F}_{xx}^{q}(L)=\langle {{\rm{\Delta }}}_{L}X{(t)}^{q}\rangle ,$$

where Δ_*L*_ *X*(*t*) = |*X*(*t* + *L*) − *X*(*t*)|. Thereby, if (cross-)correlations exist, there will be power-law relationships between the above *q*-th fluctuation functions and the scale *L* and the corresponding scaling exponents can be derived from Wang *et al*.^21^ and Barabasi *et al*.^43^ as follows,

5 $${F}_{xy}^{q}(L)\propto {L}^{q\lambda (q)}\,{\rm{and}}\,{F}_{xx}^{q}(L)\propto {L}^{q{\gamma }_{xx}(q)}.$$

Here *γ*_*xx*_(*q*) and *λ*(*q*) are the so-called generalized individual and bivariate Hurst exponents, respectively. The time interval *L* typically varies from 1 to a large positive integer *N*_1_ such that a good linear fit can be obtained in the double log plot.

If the cross-correlation is absent or not significant, then the power-law relation described by Eq. (5) no longer holds and the exponent *λ*(*q*) would be complex. To correctly quantify the level of cross-correlations, Wang *et al*.^21^ proposed the so-called AHXA coefficient as follows,

6 $$\rho (L)=\frac{{F}_{xy}^{2}(L)}{\sqrt{{F}_{xx}^{2}(L)\cdot {F}_{yy}^{2}(L)}}=\frac{\langle {\rm{sign}}({{\rm{\Delta }}}_{L}X(t)Y(t+\tau ))\cdot |{{\rm{\Delta }}}_{L}X(t)Y(t+\tau )|\rangle }{\sqrt{\langle {{\rm{\Delta }}}_{L}X{(t)}^{2}\rangle \cdot \langle {{\rm{\Delta }}}_{L}Y{(t)}^{2}\rangle }}.$$

Here *ρ*(*L*) ∈ [−1, 1] with *ρ*(*L*) = 1 indicating a perfect positive correlation and *ρ*(*L*) =  −1 indicating a perfect negative correlation. However, *ρ*(*L*) can only describe the cross-correlations at specific fluctuation range for two synchronous time series. To obtain more information on the amplitude of fluctuations contributed for the detected cross-correlations and to detect the range of cross-correlated fluctuations with potential time-delays, inspired by the idea of *q*-dependent DCCA coefficient proposed by Kwapień *et al*.^33^, we extend *ρ*(*L*) by incorporating both the time-lag factor and the fluctuation amplitude to arrive a new coefficient denoted by *ρ*_*q*_(*τ*,*L*) as follows,

7 $${\rho }_{q}(\tau ,L)=\frac{{F}_{xy}^{q}(L)}{\sqrt{{F}_{xx}^{q}(L)\cdot {F}_{yy}^{q}(L)}}=\frac{\langle {\rm{sign}}({{\rm{\Delta }}}_{L}X(t)Y(t+\tau ))\cdot |{{\rm{\Delta }}}_{L}X(t)Y(t+\tau {)|}^{q\mathrm{/2}}\rangle }{\sqrt{\langle {{\rm{\Delta }}}_{L}X{(t)}^{q}\rangle \cdot \langle {{\rm{\Delta }}}_{L}Y{(t+\tau )}^{q}\rangle }}.$$

Clearly when *q* = 2 and *τ* = 0, *ρ*_*q*_(*τ*,*L*) is exactly the AHXA coefficient *ρ*(*L*). In the case where *τ* = 0, our obtained coefficient is indeed an alternative of the *q*-dependent DCCA coefficient proposed by Kwapien *et al*. The advantage of our coefficient is that it allows us to identify the range of detrended fluctuation amplitudes that are correlated with time lags in two signals under study. Since *ρ*_*q*_(*τ*,*L*) depends on three parameters, *q*, *τ* and *L*, we call *ρ*_*q*_(*τ*,*L*) the time-lagged *q*-*L* dependent AHXA coefficient. For each fixed *q*, we can draw a *ρ*_*q*_(*τ*,*L*) surface to examine cross-correlations between two series at each time lag *τ* and each time interval *L*.
